# Control of Growth and Persistence of *Listeria monocytogenes* and β-Lactam-Resistant *Escherichia coli* by Thymol in Food Processing Settings

**DOI:** 10.3390/molecules25020383

**Published:** 2020-01-17

**Authors:** Maria Grazia Cusimano, Vita Di Stefano, Maria La Giglia, Vincenzo Di Marco Lo Presti, Domenico Schillaci, Francesco Pomilio, Maria Vitale

**Affiliations:** 1 Department of Biological, Chemical and Pharmaceutical Sciences and Technologies (STEBICEF), via Archirafi 32, 90123 Palermo, Italy; mariagrazia.cusimano@unipa.it (M.G.C.); vita.distefano@unipa.it (V.D.S.); 2 IZS Istituto Zooprofilattico Sperimentale della Sicilia via Gino Marinuzzi, 3, 90129 Palermo, Italy; maria.lagiglia8@gmail.com (M.L.G.); dimarco.vince@gmail.com (V.D.M.L.P.); maria.vitale@izssicilia.it (M.V.); 3 Centro di Referenza Nazionale Listeria at Istituto Zooprofilattico Sperimentale Abruzzo Molise, via Campo Boario, 64100 Teramo, Italy; f.pomilio@izs.it

**Keywords:** biofilm, ESBL, *Escherichia coli*, *Listeria monocytogenes*, thymol

## Abstract

The main objective of this study was to evaluate the efficacy of thymol in controlling environmental contamination in food processing facilities. The effect of thymol was tested as an agent to prevent planktonic and bacterial biofilm growth of twenty-five *Listeria* *monocytogenes* isolates from a variety of foods and five *Escherichia coli* isolates from a farm. The *E. coli* isolates were positive for extended spectrum β-lactamase (ESBL) genes. All isolates and reference strains were susceptible to thymol at Minimum inhibitory concentration (MIC) values ranging from 250 to 800 μg/mL. An interesting activity of interference with biofilm formation of *L. monocytogenes* and *E. coli* was found for thymol at sub-MIC concentrations of 200, 100, 75, and 50 μg/mL. Anti-biofilm activity ranging from 59.71% to 66.90% against pre-formed 24-h-old *L.* *monocytogenes* biofilms at concentrations of 500 or 800 µg/mL, corresponding to 2× MIC, was determined against free-living forms of six isolates chosen as the best or moderate biofilm producers among the tested strains. The property of thymol to attack *L.* *monocytogenes* biofilm formation was also observed at a concentration of 100 µg/mL, corresponding to 1/4 MIC, by using a stainless-steel model to simulate the surfaces in food industries. This study gives information on the use of thymol in food processing setting.

## 1. Introduction

Each year, millions of people get infected and die from antibiotic-resistant pathogens [1]. Antimicrobial resistance (AMR) is a serious concern for public health and its spread has increased over time due to the excessive use of similar antibiotic molecules in both human and animal health. Modern industrial agriculture is based on the extensive use of antimicrobials as therapeutics and prophylactics, particularly in intensively farmed species, such as pigs and poultry [2]. AMR renders ineffective the control of diseases caused by pathogenic bacteria that are mostly common between humans and animals [3]. It is necessary to reduce the use of antibiotics or replace them with new treatments in farms, food processing facilities, and other non-clinical settings to avoid the loss of efficacy of critically or highly important antibiotics used in clinical treatment, such as fluoroquinolones, aminoglycosides, and third- and fourth-generation cephalosporins [4].

With the aim to find alternative strategies to the use of conventional antibiotics and disinfectants on farms and industrial settings, to decrease the impact of antibiotic resistance, and to preserve the efficacy of conventional antibiotics, we focused on thymol, one of the most common terpenes found in many essential oils from a number of plants, including oregano, thymus, and myrtle. Thymol has been reported to have a wide spectrum of biological actions, such as antiseptic, antibacterial, antifungal, and anti-inflammatory activities. Currently, it is used mainly as an antiseptic, a fungicide, and a preservative and is a known ingredient in numerous complex natural antiseptic products [5]. In addition, thymol is approved by the US Food and Drug Administration (FDA) as a flavoring agent or an adjuvant and is a generally recognized as safe (GRAS) substance (https://www.accessdata.fda.gov/scripts/fdcc/?set=FoodSubstances), so it has good potential in the development of useful and sustainable alternative strategies to control pathogens in food processing settings and livestock. Moreover, the adhesion of bacteria and biofilm formation on working surfaces and food products are undesirable and harmful for the food industry and it can lead to serious health problems related to antibiotic-resistant foodborne pathogens [6]. Floors, walls, cooling pipes, freezers, packaging materials, and gloves are common sites for biofilm formation [7]. The ability to form biofilms is frequent among major foodborne pathogens, such as *Escherichia coli*, *Salmonella* spp., and *Listeria monocytogenes* [8] and biofilms remain a significant safety challenge in the food industry [9].

In recent years, many natural antimicrobials derived from plants, such as trans-cinnamaldehyde, carvacrol, eugenol, and thymol, have been tested. Interestingly, not all the tested substances with antimicrobial properties were proven to be effective against biofilms. On the contrary, some compounds (α-pinene, 1,8-cineole, (+)limonene, linalool, and geranyl acetate) enhanced biofilm growth in vitro [10]. Essential oil components from orange peels [11] and a lot of other substances, including epicatechin, β-sitosterol, and epigallocatechin from *Acacia karroo* [12] and resveratrol [13], were evaluated successfully. Thymol has been also screened as an alternative to conventional antibiotics to inhibit biofilm formation and it was found to be effective against biofilms formed by *Staphylococcus aureus* and *Staphylococcus epidermidis* [14]. The activity of thymol has also been frequently evaluated against biofilms formed by the fungal pathogen, *Candida albicans* [15].

In this study, thymol was assessed as an agent to prevent planktonic and bacterial biofilm growth of *L. monocytogenes*, which is a major cause of foodborne disease [16] and *E. coli*, which is mainly a commensal microorganism, but many pathogenic strains have been detected in humans and animals [17]. In particular, twenty-five *L. monocytogenes* isolates from a variety of food samples and five reference strains were analyzed. The interference of thymol with the growth of *L. monocytogenes* has been recognized for a long time [18,19,20], but as far as we know, it is the first time that a good number of food isolates were tested for susceptibility to thymol. The aim of the study was to suggest and support the replacement or reduction of the use of antibiotics in food processing settings and in farms.

## 2. Results and Discussion

For this study, we selected *E. coli* isolates from a swine farm with diarrhea episodes in piglets. The isolates were genetically analyzed to evaluate their virulent and pathogenic genes and extended spectrum β-lactamase (ESBL) genes. All isolates belonged to the O103 serotype and carried a hemolysin gene (*ehxA*). Four isolates carried *TEM* as the ESBL gene and three isolates carried the dispersin transporter gene (*aatA*). The *aatA* gene is a key gene in enteroaggregative *E. coli* (EAEC) and it encodes part of the outer membrane transport system (ABC transporter) involved in the translocation of the dispersin protein. The *aatA* and *astA* genes are associated with prolonged diarrhea in EAEC strains [21]. Three different phylogenetic groups were detected, although all isolates came from the same farm. Two isolates belonged to the phylogenetic group C, which is mainly associated with virulent strains in contrast to groups A and B, which are associated with commensal strains [22].

An MIC analysis of thymol was performed against planktonic forms of all five *E. coli* isolates. Thymol was active at concentrations of 400 µg/mL against four of the field isolates (4/5) and on the reference *E. coli* strains, ATCC 25922, ATCC 10536, and ATCC 8739. For the isolate *E. coli* 335, an MIC value of 300 µg/mL was detected.

The analysis on biofilm capability showed that, among the *E. coli* isolates, the best biofilm producer in microtiter plate assay at 37 °C was *E. coli* 336. This strain showed an optical density (OD) value of 0.319 at 600 nm, in contrast to the other strains that showed OD values around 0.130. We do not know why the *E. coli* strain lacking the *aatA* gene was so strong in the in vitro test, despite the association of dispersin transporter with biofilm formation. To analyze the anti-biofilm activity of thymol in *E. coli*, the experiments were run only against *E. coli* 336. Table 1 reports data concerning the genetic analysis, biofilm formation, and MIC values of thymol against planktonic forms of the field strains.

The results of thymol inhibition on the formation of new biofilms by the best biofilm producer *E. coli* 336 are presented in Table 2.

Biofilm formation by the tested strain was almost completely inhibited (94%) at a concentration of 200 μg/mL of thymol (1/2 MIC value), while it was inhibited from 59% to 54% at lower concentrations up to 50 μg/mL (1/8 MIC value). Unfortunately, no activity of thymol against pre-formed 24-h-old biofilm of *E. coli* 336 was detected. The inhibition of biofilm formation at much lower concentrations compared to MICs looks promising for further studies on the anti-biofilm properties of thymol against Gram-negative bacteria.

In *L. monocytogenes* isolates, the ability to produce biofilms ranged from 0.888 OD for the strain *L. monocytogenes* 101 (gene-serotype II), isolated from fish products, to 0.148 for the strain *L. monocytogenes* 48 (gene-serotype II), isolated from vegetable food products. The ODs of the sessile community of most of the isolates ranged from 0.461 to 0.290 and few weak producers showed ODs around 0.225. In our study, *L. monocytogenes* isolates of different origins and serotypes differed in the strength of biofilm formation and they can be divided based on their OD values into weak (OD = 0.148–0.348), moderate (OD = 0.370–0.542), and strong biofilm producers (OD = 0.553–0.888). We observed that there was no relation between gene-serotype and the ability to form a biofilm in the described experimental conditions. The best reference *L. monocytogenes* strain was LM ATCC 19114 with an OD of 0.543. The values of OD for all isolates and reference strains are reported in Table 3. Thymol was active against all planktonic forms of the tested isolates and reference strains of *L. monocytogenes* at concentrations ranging from 250 to 800 µg/mL. Ethanol, used to dissolve thymol, was not active against all the tested strains at 1% *v/v* (corresponding to the maximum volume of ethanol used at the maximum tested thymol concentration of 800 µg/mL). The MIC values against the planktonic form of L. monocytogenes and reference strains are also reported in Table 3.

**Figure 1:**
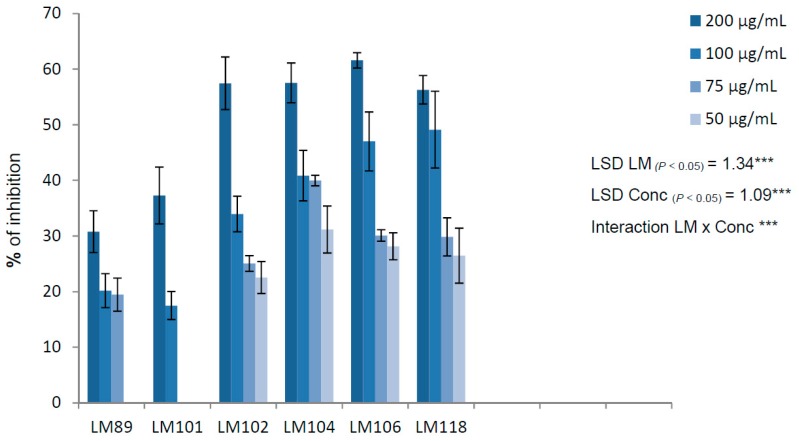
Percentages of inhibition of biofilm formation at sub-MIC concentrations of thymol against the best biofilm producers. The averages from three independent experiments are reported with the SD values. Error bars represent the SD of the data. LSD (least significant difference) was calculated at 0.05 probability level by the Student–Newman–Keuls (SNK) test [23].

To evaluate the anti-biofilm activity of thymol, we focused only on isolates from food and selected the three best biofilm producers (LM 89, LM 101, and LM 104) and three moderate producers (LM 102, LM 106, and LM 118). The results of inhibition of biofilm formation are presented in Figure 1. For each strain, the greatest inhibition was observed at the sub-MIC concentration of 200 μg/mL and the percentages of inhibition ranged from 30.78% for LM 89 to 61.56% for LM 106. A good inhibitory activity was also observed at 100 and 75 µg/mL against LM 106 and LM 118. In some cases (at a thymol concentration of 50 μg/mL for LM 89, and 75 and 50 μg/mL for LM 101), inhibition was below 15% and was not considered significant.

**Figure 2:**
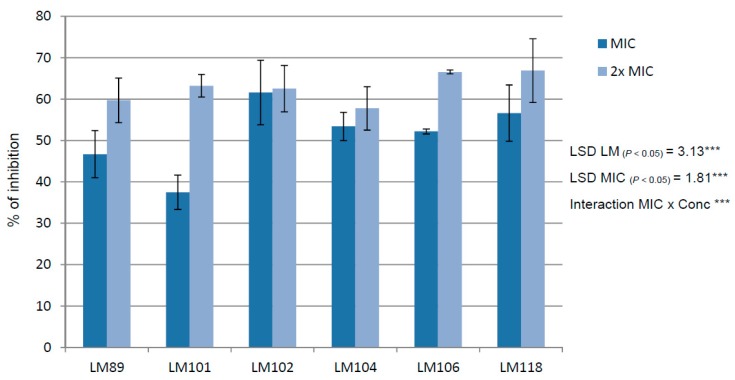
Activity against pre-formed 24-h-old biofilms at concentrations equal to MIC or 2× MIC. The averages from three independent experiments are reported with the SD values and error bars represent the SD of the data. LSD was calculated at 0.05 probability level by the SNK test [23].

The activity of thymol against mature biofilms (24-h old) of *L. monocytogenes* was evaluated at concentrations equal to MIC (250 or 400 µg/mL) or 2× MIC (500 or 800 µg/mL). The results are presented in Figure 2. At the concentration equal to the MIC value, the results against pre-formed biofilms were unevenly distributed for each isolate with values starting from 37.47% for LM 101 to 61.59% for LM 102. At the concentration equal to 2× MIC, the anti-biofilm activity was higher and similar for each strain, ranging from 59.71% to 66.90%.

**Figure 3:**
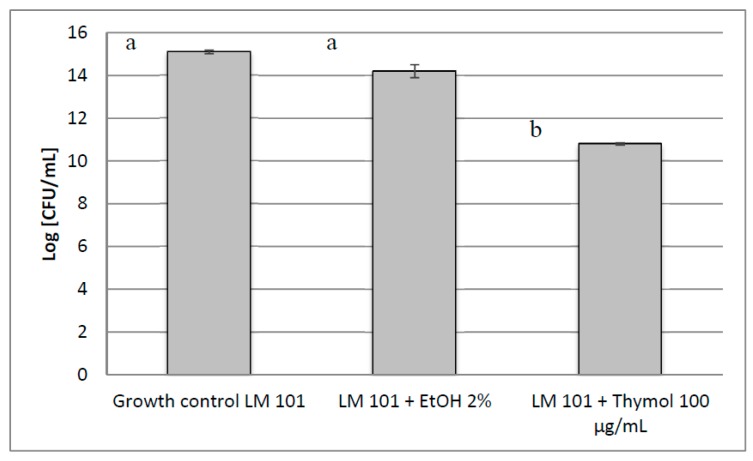
Inhibition of *L. monocytogenes* 101 (LM 101) biofilm formation on a stainless-steel surface. The averages from three independent experiments are reported with the SD values and error bars represent the SD of the data. Different letters among bars indicate statistical differences at *p* < 0.05.

The biofilm of *L. monocytogenes* 101 on a stainless-steel surface, used to simulate a common surface in food industries, was inhibited by thymol tested at a sub-MIC (1/4 MIC) concentration of 100 μg/mL. Data are reported in Figure 3. The results are expressed in terms of log Colony Forming Unit/mL (CFU/mL) of viable count by comparing the growth in the presence of thymol with a non-treated control and another control treated with ethanol at the same concentration (2% *v*/*v)* used to dissolve thymol. We observed a considerable log reduction (4.3) in the presence of thymol. Ethanol elicited a log reduction of 0.9.

The search for alternative molecules to control bacterial infections and contamination is an important challenge worldwide, especially in the era of antimicrobial resistance. In this paper, we focused on two important bacterial species, a Gram-positive bacterium (*L. monocytogenes)* and a Gram-negative bacterium (*E. coli*), to test the antimicrobial activity of thymol in both the planktonic and sessile forms of these microorganisms. The analysis of the activity of thymol on the planktonic forms showed MIC values ranging from 250 to 400 μg/mL for the food isolates of *L. monocytogenes* and 300 to 400 μg/mL for the virulent and resistant *E. coli* isolates from the farm; however, lower concentrations are necessary to inhibit biofilm formation of both species and suggesting the possibility to develop molecules from thymol to inhibit both Gram-positive and Gram-negative bacteria. The inhibitory activity toward biofilm organization of both Gram-positive and Gram-negative bacteria could be very important in the sanitizing aspect to avoid persistent contamination. It is known that conventional sanitizers and detergents are more efficient against planktonic cells than against biofilms, and *L. monocytogenes* biofilm is still a challenge due to its extraordinary ability to survive in many drastic conditions [24]. The search for alternative methods to inhibit biofilm formation by *L. monocytogenes* is an important task to tackle the spread of antimicrobial resistance. We found that sub-MIC concentrations were able to inhibit biofilm formation on polystyrene microtiter plates at 37 °C. In some isolates (LM 102 and LM 104), inhibition was observed at levels as low as 1/8 MIC. Similar results were also observed with *E. coli* 336.

The anti-adhesion property of thymol could play an important role against foodborne pathogens, such as pathogenic *E. coli* and *L. monocytogenes*, suggesting and confirming that thymol could be used in the food industry as an environment-friendly antimicrobial agent to prevent or eradicate microbial biofilms [25,26]. The results of inhibition on the stainless-steel surface, simulating a food processing equipment, against the best biofilm producer *L. monocytogenes* (LM 101) further supported the potential use of thymol in sanitation procedures of food processing equipment.

Finally, the replacement or reduction of the use of conventional antimicrobials in food processing settings and in intensive farms by using a well characterized and sustainable molecule such as thymol can contribute to tackling the AMR phenomenon and to preserve antibiotics that should be reserved for human medicine.

## 3. Materials and Methods

### 3.1. Bacterial Strains

The following strains of *L. monocytogenes* were used: Twenty-five isolates from different foods (meat, fish, vegetable, and dairy products) and belonging to gene-serotype II or IV were supplied by the Italian Reference Laboratory (Teramo, Italy) for *Listeria monocytogenes*, Istituto Zooprofilattico Sperimentale of Abruzzo and Molise, Teramo, Italy. Five *L. monocytogenes* reference strains were also used (ATCC 7644, ATCC 19114, ATCC 19115, NCTC 10887, and NCTC 18890). Five *E. coli* strains (334, 335, 336, 337, and 339) isolated from a swine farm with diarrhea episodes and supplied by Istituto Zooprofilattico Sperimentale of Sicilia, Palermo, Italy and three *E. coli* reference strains (ATCC 25922, ATCC 10536, and ATCC 8739) were also included in the analysis. The media used in this study were tryptic soy broth (TSB; Sigma-Aldrich, Milan, Italy) with 2% glucose and tryptic soy agar (TSA) without glucose. Thymol (purity ≥ 98.5%) was purchased from Sigma-Aldrich, Milan, Italy.

### 3.2. Genetic Analysis on E. coli Isolates

The animal isolates of *E. coli* were analyzed through multiplex PCRs for the presence of extended spectrum β-lactamase (ESBL) and virulence factor genes as described in [27,28].

The following multiplex PCRs were performed:

Two multiplex PCR assays (Set 1 and Set 2) for the genes of ESBL

Set 1 for *TEM*, *SHV*, *CTX M*, and *OXA* genes;

Set 2 for *CTX MI*, *CTX MII*, *CMYII*, and *DHA* genes.

Two multiplex PCR assays (MPX1 and MPX2) for virulence factors and specific serogroups:

MPX1 for serogroups O45, O145, O26, and O157;

MPX2 for serogroups O103, O121, and O111.

A phylogroup analysis was performed as described in [29]. For the phylogroups A, B1, B2, and D, a multiplex PCR was set to detect the genes *chuA*, *yjaA*, *TspE4*, and *arpA*. For the phylogroups C and E, single PCR assays were performed (*trpAgpC.1*/*trpAgp*C.2 for group C and *arpAgp*E.f/*ArpAgp*E.r for group E).

All PCR reactions were performed in 25 μL final volume with 2 units of Taq Gold polymerase (Life Technologies, Monza, Italy). The results were visualized on 2% agarose gels with SYBR^®^ Safe DNA gel stain under UV light.

### 3.3. Determination of Minimal Inhibitory Concentrations (MICs)

The MICs of thymol were determined by an agar dilution method using tryptic soy agar (TSA). A suitable volume of a solution of thymol in ethanol (ranging from 800 to 125 μg/mL) was added to 20 mL of molten medium to obtain the required concentrations. The resulting mixture was poured onto Petri plates and allowed to solidify. Two plates were included as growth controls—one without the antimicrobial agent and the other with the maximum concentration of ethanol—to exclude the activity of ethanol at the highest concentration. The plates were inoculated with bacterial suspensions from a 24-h culture containing approximately 10^6^ colony forming units /mL (CFU/mL) of each strain and incubated at 37 °C overnight. MICs of thymol were recorded as the lowest concentration of thymol that completely inhibited bacterial growth (absence of colonies). Ethanol, used to dissolve thymol, did not show any antibacterial activity at the concentration of 1% *v*/*v* used at the maximum tested thymol concentration of 800 µg/mL. Two replicates were tested every time and the tests were repeated at least in three independent experiments. The MIC values obtained with the agar dilution method described above were also confirmed by a broth dilution micro-method using tryptic soy broth (TSB) in a 96-well plate [28].

### 3.4. Evaluation of Biofilm Formation and Inhibition

Test tubes filled with 5 mL TSB containing 2% glucose were inoculated with a loopful of a 24-h culture from TSA tubes and incubated at 37 °C for 24 h. After the incubation time, 200 μL of the medium (TSB with 2% glucose) and 2.5 μL of bacterial suspension with an optical density (OD) of about 0.040 at 570 nm, corresponding to approximately 10^6^ CFU/mL, was added to each well. The microtiter plate was sealed with parafilm and incubated at 37 °C for 24 h. The wells were washed twice with 0.9% sterile NaCl and stained with 100 μL of 0.1% crystal violet solution for 10 min at room temperature. The excess solution was removed and the plate was washed twice using tap water. To solubilize the dye, 200 μL of ethanol was added to each stained well for 10 min at room temperature [30]. All tests involved six replicates and were repeated at least in three independent experiments. Optical density (OD) was read at a wavelength of 600 nm using a plate reader (GloMax^®^-Multi Detection System, Promega Italia s.r.l, Milan, Italy). The calculation of mean and standard deviation (SD) was done on a PC with the computer program, Microsoft Excel 2010 (Microsoft Corporation, Redmond, WA, USA).

### 3.5. Inhibition of Biofilm Formation

*L. monocytogenes* or *E. coli* strains were incubated as described above. An aliquot of 2.5 μL was added to each well of a polystyrene, sterile, flat-bottomed 96-well plate filled with 200 μL of the medium (TSB with 2% glucose). Sub-MIC concentrations of thymol were added (200, 100, 75, and 50 μg/mL) and each concentration was added to at least three wells. After biofilm growth, the content of each well was removed and the plate was washed and stained with crystal violet and re-dissolved as already described for evaluation of biofilm formation. The OD was measured at a wavelength of 600 nm using a plate reader (GloMax^®^-Multi Detection System). The experiments were run at least in triplicates and three independent experiments were performed. To calculate the percentages of inhibition the following formula was used:

(1) $$\%\;of\;Inhibition=\;\frac{OD\;growth\;control-OD\;sample}{OD\;growth\;control}\;\times \;100\%.$$

The calculation of mean and SD was done on a PC with the computer program, Microsoft Excel 2010.

### 3.6. Anti-Biofilm Activity

The medium from each well of the 24-h-old biofilm was removed and the plate was washed and left to dry. Aliquots of 200 μL of fresh medium (TSB with 2% glucose) were added to each well followed by aliquots of thymol to reach concentrations equal to MIC and 2× MIC obtained against the planktonic forms of the tested strains. The microtiter plate was sealed with parafilm and incubated at 37 °C for 24 h. The content of each well was removed and the plate was washed twice with 0.9% sterile NaCl (200 μL to each well) and left to dry. The staining procedure was performed as described above. The OD was measured at a wavelength of 600 nm using the plate reader (GloMax^®^-Multi Detection System). The experiments were run at least in triplicates and three independent experiments were performed. The percentages of inhibition can be calculated with Equation (1) as described above.

### 3.7. Inhibition of Biofilm Formation on Circular Stainless-Steel Coupons Using the Viable Plate Count Method

In a 24-well plate, circular sterile stainless-steel coupons (14–15 mm diameter with 1.0 mm thickness) were put as the flat-bottom in each well. Each well was filled with 2 mL of TSB with 2% glucose and a sub-MIC concentration (100 μg/mL) of thymol was added. An aliquot of 25 μL from a suspension of *L. monocytogenes* LM 101 with OD of about 0.040 at 570 nm, corresponding to approximately 10^6^ CFU/mL, was added to each well. After incubation for 24 h, the content of each well was removed and the bottom surface was washed twice with 0.9% sterile NaCl (2 mL to each well). The inoculum scraped from the stainless-steel surface was put in a test tube with 10 mL of NaCl (0.9% *w*/*v* solution) and sonicated (ultrasonic nominal power equal to 215 kHz) for 2 min. A suitable number of 10-fold dilutions were prepared and 100 μL aliquots of each dilution were plated in TSA [31]. Plates were then incubated at 37 °C and CFU/mL were counted after 24 h and compared to CFU/mL formed on growth control wells without inhibitor and growth control wells with 2% *v*/*v* ethanol used to dissolve thymol. All tests involved two replicates and were repeated at least in three independent experiments. The activity was reported in terms of log reduction of viable plate counts [24]. The calculation of mean and SD was done on a PC with the computer program, Microsoft Excel 2010.

### 3.8. Statistical Analysis

Data were analyzed using the Bartlett’s test for homogeneity of variances and analysis of variance (ANOVA). Means were statistically separated on the basis of Student–Newman–Keuls test, when the F-test of ANOVA for treatment was significant at least at the 0.05 probability [23].

## Figures and Tables

**Table 1:** Genetic analysis on *Escherichia coli* isolates, biofilm formation, and thymol MIC data.

										*aatA*	*shf*	*Irp2*	*wzxO103*	*ehxA*		Thymol
*E. coli Isolate*	Phylogenetic Group	*TEM*	*CTX MIV*	*OXA*	*SHV*	*CTX MI*	*CMYII*	*CTX MII*	*DHA*	Dispersin Transporter	*Cryptic Open Reading*	*Yersinia* Bactin	O-polysaccharide Export	Virulence Gene	Biofilm Formation	MICs (µg/mL)
334	A	+	−	−	−	−	−	−	−	−	−	−	+	+	−	400
335	B1	+	−	−	−	−	−	−	−	+	−	−	+	+	−	300
336	B1	+	−	−	−	−	−	−	−	−	−	−	+	+	+	400
337	C	+	−	−	−	−	−	−	−	+	−	−	+	+	−	400
339	C	+	−	−	−	−	−	−	−	+	−	−	+	+	−	400

**Table 2:** Inhibition of *E. coli* 336 biofilm formation at sub-MIC concentrations of thymol. The averages from three independent experiments are reported with the SD values.

Percentage Inhibition Biofilm Formation				
Thymol	200 μg/mL	100 μg/mL	75 μg/mL	50 μg/mL
Inhibition %	94% ± 4.0%	59.4% ± 1.6%	55% ± 1.0%	54.5% ± 3.5%

**Table 3:** Evaluation of biofilm formation as the optical density (OD) of crystal violet-stained adherent biomass and thymol MIC values in µg/mL against *L. monocytogenes* isolates and reference strains. The averages from three independent experiments are reported with the SD values. Different letters indicate statistical differences at *p* < 0.05 among the *L. monocytogenes* isolates.

*L. monocytogenes*	Origin Product	Thymol MIC (µg/mL)	OD
45	Meat	250	0.424 ± 0.008 efg
46	Meat	250	0.447 ± 0.04 ef
47	Vegetable	250	0.228 ± 0.03 m
48	Vegetable	400	0.148 ± 0.02 n
49	Vegetable	400	0.593 ± 0.08 c
50	Vegetable	400	0.376 ± 0.05 ghij
51	Vegetable	400	0.520 ± 0.06 d
52	Vegetable	400	0.290 ± 0.04 l
53	Dairy	400	0.388 ± 0.03 ghi
79	Dairy	400	0.388 ± 0.04 ghi
85	Meat	400	0.386 ± 0.01 ghi
89	Meat	400	0.663 ± 0.07 b
96	Dairy	400	0.225 ± 0.03 m
98	Dairy	400	0.348 ± 0.003 ijk
99	Fish	400	0.461 ± 0.04 e
100	Fish	400	0.323 ± 0.006 k
101	Fish	400	0.888 ± 0.12 a
102	Fish	400	0.536 ± 0.03 d
103	Fish	400	0.598±0.03 c
104	Fish	400	0.553 ± 0.05 d
105	Dairy	400	0.370 ± 0.05 hij
106	Dairy	250	0.537 ± 0.06 d
107	Fish	250	0.414 ± 0.05 fgh
112	Meat	250	0.335 ± 0.03 jk
118	Dairy	250	0.542 ± 0.02 d
ATCC 7644		800	0.424 ± 0.06 efg
ATCC 19114		800	0.543 ± 0.07 d
ATCC 19115		800	0.252 ± 0.02 lm
NCTC 10887		800	0.405 ± 0.04 fgh
NCTC 18890		800	0.251 ± 0.02 lm
LSD (*p* < 0.05)			0.033
